# Comparison of independent screens on differentially vulnerable motor neurons reveals alpha-synuclein as a common modifier in motor neuron diseases

**DOI:** 10.1371/journal.pgen.1006680

**Published:** 2017-03-31

**Authors:** Rachel A. Kline, Kevin A. Kaifer, Erkan Y. Osman, Francesco Carella, Ariana Tiberi, Jolill Ross, Giuseppa Pennetta, Christian L. Lorson, Lyndsay M. Murray

**Affiliations:** 1 Centre for Integrative Physiology, University of Edinburgh, Edinburgh, United Kingdom; 2 Euan McDonald Centre for Motor Neuron Disease Research, University of Edinburgh, Edinburgh, United Kingdom; 3 Bond Life Sciences Center, University of Missouri, Columbia, Missouri, United States of America; 4 Department of Veterinary Pathobiology, College of Veterinary Medicine, University of Missouri, Columbia, Missouri, United States of America; The Jackson Laboratory, UNITED STATES

## Abstract

The term “motor neuron disease” encompasses a spectrum of disorders in which motor neurons are the primary pathological target. However, in both patients and animal models of these diseases, not all motor neurons are equally vulnerable, in that while some motor neurons are lost very early in disease, others remain comparatively intact, even at late stages. This creates a valuable system to investigate the factors that regulate motor neuron vulnerability. In this study, we aim to use this experimental paradigm to identify potential transcriptional modifiers. We have compared the transcriptome of motor neurons from healthy wild-type mice, which are differentially vulnerable in the childhood motor neuron disease Spinal Muscular Atrophy (SMA), and have identified 910 transcriptional changes. We have compared this data set with published microarray data sets on other differentially vulnerable motor neurons. These neurons were differentially vulnerable in the adult onset motor neuron disease Amyotrophic Lateral Sclerosis (ALS), but the screen was performed on the equivalent population of neurons from neurologically normal human, rat and mouse. This cross species comparison has generated a refined list of differentially expressed genes, including CELF5, Col5a2, PGEMN1, SNCA, Stmn1 and HOXa5, alongside a further enrichment for synaptic and axonal transcripts. As an *in vivo* validation, we demonstrate that the manipulation of a significant number of these transcripts can modify the neurodegenerative phenotype observed in a *Drosophila* line carrying an ALS causing mutation. Finally, we demonstrate that vector-mediated expression of alpha-synuclein (SNCA), a transcript decreased in selectively vulnerable motor neurons in all four screens, can extend life span, increase weight and decrease neuromuscular junction pathology in a mouse model of SMA. In summary, we have combined multiple data sets to identify transcripts, which are strong candidates for being phenotypic modifiers, and demonstrated SNCA is a modifier of pathology in motor neuron disease.

## Author summary

The term “motor neuron disease” refers to a group of disorders, causing progressive paralysis of affected patients due to the degeneration of motor neurons cells which control voluntary movements. Importantly, not all motor neurons appear to be affected in the same way, with those that control the face being affected less that those that control the abdomen. The reason why some motor neurons are more vulnerable is unknown; however, understanding this may provide new targets for therapeutics to slow motor neuron degeneration either as stand-alone therapeutics or in combination with SMN-inducing compounds. In this study, we analysed gene expression in different groups of motor neurons and compared this to previously published expression data to identify commonalities. One of the common transcripts was alpha-synuclein (SNCA), which was consistently expressed at lower levels in vulnerable motor neurons. Importantly, when SNCA levels were increased in a mouse model of motor neuron disease, the disease phenotype was significantly reduced, including an extension in survival and reduction in motor neuron pathology. Collectively, these results demonstrate that this approach can identify disease modifiers that can reduce disease severity in models of motor neuron disease and potentially identify new therapeutic targets.

## Introduction

The term “motor neuron disease” refers to a group of disorders in which motor neurons are a prominent pathological target. Such disorders are generally severely disabling and frequently fatal within months to years of diagnosis. Effective treatments for many motor neuron diseases are currently lacking. Motor neuron diseases can be categorized into various types. For example, Amytrophic Lateral Sclerosis (ALS) affects upper and lower motor neurons and disease onset is typically in adulthood between the ages of 30 and 50. While approxitamely 10% of ALS cases are familial, the majority of new cases are sporadic[1]. Spinal Muscular Atrophy (SMA) refers to a type of motor neuron disease that is caused by homozygous loss of the *SMN1* gene[2, 3], resulting in the loss of lower motor neurons. Due to the presence of an additional partially functional copy of *SMN*, termed *SMN2*, which can exist in a range of copy numbers, SMA severity can vary widely[4]. However, the most common form of this disease has an onset of less than 6 months of age and a life expectancy of under 2 years without significant respiratory support. Spinal and Bulbar Muscular Atrophy (SBMA) is an X-linked motor neuron disease caused by an expansion of a trinucleotide repeat in the androgen receptor gene[5]. SBMA also appears to result in the degeneration of lower motor neurons with onset between 30 and 50 years of age. SBMA disease progression is typically slower than other types of motor neuron diseases and patients typically have normal life expectancies.

Distinct motor neuron diseases with their own specific cause, onset and prognosis are united by the common vulnerability and loss of motor neurons. Importantly, however, in each disease motor neurons are not uniformly vulnerable. In both patients and animal models, some motor neuron populations are lost very early in the disease, whilst others remain remarkably intact, even at late stages of disease. For example, in SMA the pattern of motor neuron pathology is highly predictable. This has been extensively characterised in mouse models of the disease[6–9]. The pattern of selective vulnerability in patients is less-well documented but it has been described as highly stereotyped, even within muscles groups[10]. One of the last groups of motor neurons to be affected are those supplying the muscles of the face, in particular those supplying the extra-ocular muscles[11]. The location of disease onset in patients with ALS is more variable; however, there appears to be a sparing of the motor neurons which supply the extra-ocular muscles[12]. This finding has been corroborated in mouse models of ALS in which there appears to be a marked differential vulnerability of specific cranial nerve nuclei[13, 14]. Therefore, despite subtleties in the different patterns of selective vulnerability between different motor neuron diseases, there are also important pathologenic similarities. This point was highlight in a study by Comley et al., demonstrating a shared pattern of selective vulnerability in mouse models of ALS and SMA [6]. Recent work has also shown significant overlap in the molecular mechanisms which govern distinct subtypes of motor neuron diseases [15]. Identifying the common mechanisms giving rise to selective protection or vulnerability of motor neurons will provide important biological insight into motor neuron development, but can also lead to the identification of novel therapeutic targets for motor neuron diseases.

This observed selective vulnerability of motor neurons creates a valuable opportunity to investigate the mechanism of motor neuron vulnerability in motor neuron diseases. Indeed, we have recently utilised this observation to investigate the transcriptional differences occurring pre-symptomatically in a mouse model of SMA[16]. In this study, motor neuron vulnerability was defined by the level of pathology observed at the neuromuscular junction (NMJ). Pathology at the NMJ was defined as denervation, pre-synaptic swelling and decrease in pre- and post-synaptic complexity. We identified an increase in vulnerability in the NMJs from the abdominal muscles compared to those in the cranial muscles. The motor neuron cell bodies which corresponded to these differentially vulnerable NMJs were isolated and RNAseq was performed to generate transcriptional profiles for abdominal and cranial motor neurons from SMA and WT mice. The purpose of this study was to identify transcriptional changes which correlate with a decrease in Smn levels, and those which correlate with an increase in motor neuron pathology. However, an additional benefit of this screen was to profile the transcriptomes of vulnerable and resistant motor neurons from wild-type mice. We suggest that genes which are differentially regulated between these two populations of healthy motor neurons have the potential to be important modifiers of disease. Indeed, identifying the modifiers in selectively resistant motor neuron pools which are responsible for their decreased their vulnerability could provide key insight for the development of strategies to protect more vulnerable motor neurons.

This idea has previously been exploited by a number of independent groups who have observed predictable patterns of selective vulnerability in different motor neuron diseases, and aimed to identify transcriptional changes between vulnerable and resistant motor neuron pools[17–19]. Each of these studies identified motor neurons which were predictably vulnerable or resistant in SMA, SBMA or ALS patients or animal models, and used laser capture microdissection to isolate these equivalent motor neurons from neurologically healthy humans, wild-type rats or mice. These 3 screens have identified a large number of transcriptional changes between differentially vulnerable motor neurons in healthy individuals.

The transcriptional profiles from these screens, therefore, represent a valuable set of data, detailing expression changes occurring between vulnerable and resistant motor neurons from a range of species and ages, all from healthy individuals. Such changes cannot, therefore, be due to any pathology, and are rather reflective of instrinsic differences between motor neuron pool which may alter their vulnerability to pathological situations. In our search for transcriptional modifiers of motor neurons, we suggest that common features between these transcriptional changes have a high chance of being modifiers of motor neuron pathology. Features which are common across transcriptional screens are also likely to be modifiers across multiple motor neuron diseases, rather than just one MND subtype. Gaining knowledge of these modifiers will give insight into shared mechanism of disease, and therefore potential shared therapeutic options.

In this study, we compared the transcriptome of vulnerable (innervating abdominal muscles) and resistant (innervating cranial muscles) motor neurons from P10 wild-type mice which are differentially vulnerable in mouse models of SMA. In order to refine this data set, we reanalyzed the raw data from 3 published independent microarray screens on healthy but differentially vulnerable neurons and compared it to our RNAseq data. We identified 6 transcripts that share common directional changes in all 4 screens: CELF5, Col5a2, PGEMN1, SNCA, STMN1 and HOXa5. Functional clustering of the transcripts that were changed in 2 or more of the 4 screens revealed an enrichment for synaptic and axonal transcripts. Introduction of the differentially expressed genes into a *Drosophila* model of ALS8 rescued hallmarks of the neurodegenerative phenotype, demonstrating that the differentially expressed genes can function in disease-relevant pathways. Due to the lack of Drosophila homologue for SNCA, and because of evidence from the literature implying SNCA may have neuroprotective qualities, we investigated whether increasing levels of SNCA could amleriorate the phenotype in a mouse model of motor neuron disease. ScAAV9-*SNCA* was delivered to a mouse model of SMA, resulting in a significant decrease in disease severity, including an extension in survival and increased weight gain. Importantly, NMJ pathology in scAAV9-*SNCA* treated mice was significantly improved, providing evidential support for the notion that differentially expressed genes from susceptible motor neurons can serve as disease modifiers.

## Results

### Differential levels of neuromuscular junction pathology correlate with differential expression levels of a large number of transcripts

**Fig 1:**
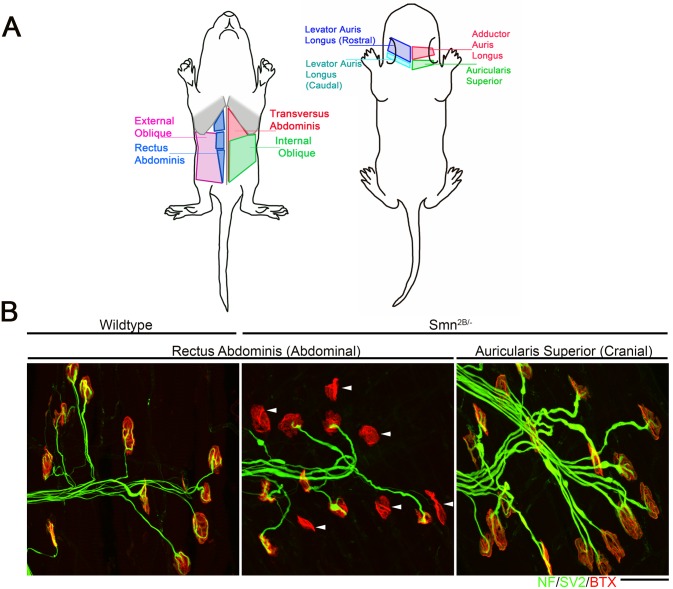
Intermuscular variability in the levels of neuromuscular junction pathology in the Smn^2B/-^ mouse model of SMA. A) Schematic diagram showing location of muscles which were innervated by either vulnerable or resistant motor neurons in a mouse model of SMA. Vulnerable muscles, as defined by increased neuromuscular junction (NMJ) pathology, include: external and internal oblique; transversus abdominis; and rectus abdominis. Resistant muscles, as defined by a low level of NMJ pathology, include: levator auris longus; auricularis superior; and adductor auris longus. B) Confocal micrographs showing NMJs with the pre-synaptic terminal labeled with antibodies against neurofilament (NF; Green) and synaptic vesicle protein 2 (green) and the muscle endplate labeled with alpha-bungarotoxin (red) from rectus abdominis and auricularis superior muscles. Note that in the wild-type abdominal and *Smn*^*2B/-*^ cranial muscle, all endplates appear fully innervated where each endplate is covered by the pre-synaptic terminal labeled with SV2 and NF. In the rectus abdominis from the *Smn*^*2B/-*^, mouse there is evidence of significant NMJ pathology, as evidenced by endplates lacking a pre-synaptic terminal (white arrow heads). Scale bar = 40μm.

Differentially vulnerable motor neurons have been reported in patients and in mouse models of SMA [6–11]. In the *Smn*^*2B/-*^ SMA mouse model, selective vulnerability can be observed at the neuromuscular junction (NMJ). Analysis of NMJs in the abdominal muscles revealed a high level of denervation, alongside other markers of pathology such as neurofilament accumulation, shrinkage of endplates and a decrease in endplate complexity (Fig 1)[8, 16]. This represents a “vulnerable” population. Analysis of a group of cranial muscles, which are innervated by motor neurons residing in the facial nucleus of the brainstem, show no evidence of denervation and minimal evidence of other markers of NMJ pathology and therefore represent a “resistant” population (Fig 1) [8, 16].

In previous work, we used intramuscular injection of dextran molecules to trace the motor neurons’ cell bodies which correspond to these differentially vulnerable groups of NMJs[16]. Cell bodies were isolated by laser capture microdissection and RNAseq was performed on extracted RNA. Parallel experiments were performed on wild-type and *Smn*^*2B/-*^ SMA mice. This study[16] focused on the differences between SMA and WT mice at a pre-symptomatic time point (P10). However, this work also produced a transcriptional profile of thoracic (vulnerable) and cranial (resistant) motor neurons from wild-type mice. In the current study, we address the hypothesis that the transcriptional changes occurring between vulnerable and resistant motor neurons in wildtype mice reflect intrinsic differences which contribute to the differential vulnerability observed in the mouse model of disease. Comparison of the transcriptional data between resistant and vulnerable motor neurons from wild-type mice resulted in 910 significantly altered transcripts with a fold change of >1.5 fold, with 218 up-regulated and 692 down regulated in vulnerable versus resistant motor neurons (Table 1, S1 Table). Functional clustering of these transcriptional changes using DAVID bioinformatics resources version 6.8 revealed an enrichment for extracellular matrix and glycoproteins (Table 2).

**Table 1:** Top 20 transcriptional changes identified between vulnerable (abdominal) and resistant (cranial) motor neurons from P10 wild-type mice.

Gene Symbol	Log Fold Change	Q Value	Gene Symbol	Log Fold Change	Q Value
Hoxc8	11.1	9E-06	Ptgds	-11.0	0E+00
Hoxd8	10.4	1E-03	Tbx15	-10.0	9E-05
Hoxc9	7.8	6E-04	Glis1	-9.7	3E-04
Hoxa5	7.1	1E-07	Slc6a4	-9.6	1E-03
D930020B18Rik	6.8	1E-02	Tph2	-9.4	1E-06
Npy5r	6.8	2E-03	Omd	-9.2	5E-02
BC023105	6.5	5E-03	Fam180a	-8.9	9E-06
AU021034	6.3	5E-04	Gm15605	-8.8	1E-02
AV039307	6.2	2E-05	Cdh1	-8.7	2E-09
Mstn	5.6	1E-04	Insm1	-8.6	9E-05
Hoxb7	5.5	3E-05	Nov	-8.5	1E-13
Gm20520	5.4	2E-02	Moxd1	-8.5	3E-05
BC037032	5.4	1E-03	Slc17a7	-8.2	8E-05
Hoxd9	5.4	5E-03	Ogn	-8.1	0E+00
Troap	5.3	2E-02	Gal	-7.9	1E-04
Prss56	5.3	4E-02	Gxylt2	-7.6	5E-03
Thpo	5.2	2E-04	Thbd	-7.5	2E-13
Mpz	5.2	2E-04	Trim58	-7.4	9E-11
Gm5077	5.0	3E-03	Cyp2f2	-7.3	4E-02
Glp1r	5.0	4E-04	Aldh1a2	-7.2	7E-11

**Table 2:** Top 5 functional clustering of transcriptional changes identified between vulnerable and resistant motor neurons.

Functional Cluster	Enrichment Score
Extracellular Matrix	22.8
Glycoprotein/Extracellular Region	22.1
Cell adhesion	9.04
Focal Adhesion	6.53
Pattern binding	5.86

### Comparison of RNAseq data with published screens refines list of potential modifiers

This screen has identified a large number of transcriptional changes. However, it is difficult to differentiate those which merely correlate with differential vulnerability from those which actually contribute to differential vulnerability. In order to identify those changes which had the highest probability of being modifiers and perhaps play also a causative role in motor neuron pathology, we compared the results of our screen with the results of other screens on different populations of differentially vulnerable motor neurons which have been previously published (Table 3).

**Table 3:** Summary of independent screens on differentially vulnerable motor neurons.

Paper	Differentially vulnerable in	Vulnerable	Resistant	Source
Hedlund *et*. *al*. 2010 [18]	SMBA/ALS/SMA	Cervical MNs	Occulomotor/ Abducens nucleus	Wild-type adult rat
Brockington et al., 2013 [17]	ALS	Cervical MN	Occulomotor Nucleus	Neurologically normal human (59–67 YOA)
Kaplan et al., 2014 [19]	ALS	Lumbar spinal cord (L5)	Occulomotor Nucleus	P7 wild-type mice

**Fig 2:**
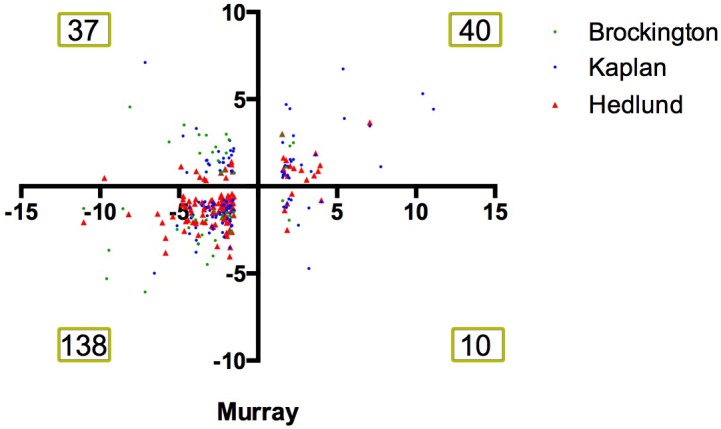
Comparison of 4 independent screens on differentially vulnerable motor neurons reveals a large number of common transcriptional changes. Scatter plot showing the fold change of transcripts which were differentially expressed in differentially vulnerable motor neurons in the RNAseq performed by Murray et al., 2015 [16] and in the microarray study on differentially vulnerable motor neuron performed by Brockington et al., 2013 [17] (green), Kaplan et al., 2014 [19] (blue) and Hedlund et al., 2010 [18](red). Numbers denote number of number of common transcriptional screens within each quadrant of the plot. Note that the majority of changes occur with a common directional change i.e. fall within the bottom left or top right quadrant of the graph.

Raw microarray data were re-analysed and a list of differentially expressed genes for each screen was generated. As screens were performed in different species, genetic homologues were identified, and all genes were listed corresponding to the mouse official gene symbol. Comparison of the results of the 3 microarray studies with our own RNAseq data revealed a large number of common changes, with the majority occurring the same direction of change (Fig 2). Transcriptional changes from these 4 transcriptional screens were sorted based on direction of change. This resulted in the identification of 595 transcripts which were altered in a common direction in 2 screens (S2 Table), 62 transcripts which were common in 3 screens (S3 Table) and 6 transcripts which were common in all 4 screens (Table 4). Functional clustering of the transcriptional changes occurring in 2 or more screens revealed an enrichment for axonal and synaptic proteins (Table 5).

**Table 4:** Transcriptional changes which are common across all 4 screens on differentially vulnerable motor neurons.

	Fold Change			
	Brockington	Kaplan	Murray	Hedlund
Celf5	-3.3	-1.4	-2.0	-2.7
Col5a2	-3.1	-3.8	-3.9	-2.1
Pgrmc1	-1.5	-0.8	-1.6	-2.6
Snca	-1.3	-1.1	-1.8	-2.0
Stmn1	-1.4	-0.8	-1.6	-1.0
Hoxa5	2.9	3.5	7.1	3.7

**Table 5:** Functional clustering of transcriptional changes which are common in 2 or more of the screens on differentially vulnerable motor neurons.

Functional Cluster	Enrichment Score
Neuron projection/Axon	8.00
Synapse/Synaptic Vesicle/Cell junction	7.80
Synaptic transmission	3.91
Homeobox/DNA binding	3.89
Extracellular Matrix	3.54
Ionic Channel	3.52
Synaptic vesicle	3.21
Membrane Fraction	3.08
Clathrin Coated Vesicle	2.93
Glycoprotein	2.91

### Differentially expressed transcripts modify the degenerative eye phenotype in a Drosophila model of ALS8

To determine whether the identified transcripts can modify neurodegenerative pathways, a *Drosophila* model of ALS was used to functionally validate the candidate genes by driving expression of the candidate genes or by transiently knocking-down expression. The purpose of this screen was to determine whether a significant number of transcripts identified here were capable of modifying the phenotype in an independent model of neurodegeneration induced by an ALS causing mutation. Transcripts were selected that either changed in 3 or more of the transcriptional screens (Table 4 and S2 Table) or were featured in one of the two top functional clusters (axonal or synaptic transcripts) (Table 5). In this model, a *Drosophila* line expresses the P58S mutation in the VAMP associated protein B gene (VAPB). This is equivalent to the P56S mutation in human VAMP which is a causative mutation of human motor neuron disease, including ALS8 [20]. This mutation has been shown to affect a range of cellular processes which have been implicated in MND, including the unfolded protein response, endocytosis, vesicular trafficking, mitochondrial defects and autophagy [21–26]. The *Drosophila* homologue of VAPB is termed VAP-33-1, or DVAP. In previous work, DVAP-P38S expression was driven in the eye of *Drosophila* using the UAS/GAL4 system[27], with an *eyeless-GAL4* (*ey-GAL4*) driver. This resulted in a roughness of the adult Drosophila eye and a significant reduction its size, which could be attributed to a decrease cell survival[28]. This model and the eye phenotype readout has previously been used in a large-scale enhancer and suppressor screen for genetic modifiers of ALS8 pathology [23].

As outlined above, for this *in vivo* validation, we chose to include all transcripts which were changed in 3 or more screens, as well as those pertaining to the top two functional clusters, of axonal or synaptic transcripts. This resulted in a list of 160 transcripts. *Drosophila* homologues were predicted for each transcript using the DRSC Integrative Ortholog Prediction tool (http://www.flyrnai.org/cgi-bin/DRSC_orthologs.pl). Results were filtered to return only the best match where more than one homologue was found and restricted to those with a DIOPT score of greater than 2. Where more than one homologue was found with an equivalent weighted score, all potential homologues were included. From the 160 transcripts listed, we identified an homologue for 116, which includes 39 transcripts up-regulated and 77 down regulated transripts in vulnerable motor neurons.

For transcripts which were up-regulated in vulnerable motor neurons, we identified publically available lines carrying RNAi constructs to knock down transcripts of interest. For the majority of lines (38/46), we selected those with zero off target effect predicted. For a small number of lines this was not possible. In this case, lines with the minimum number of off target effects were used. For transcripts which were down-regulated in vulnerable motor neurons, we identified publically available lines carrying a P-element insertion which, based on the position and orientation, would be predicted to result in an over expression of the transcripts of interest.

**Fig 3:**
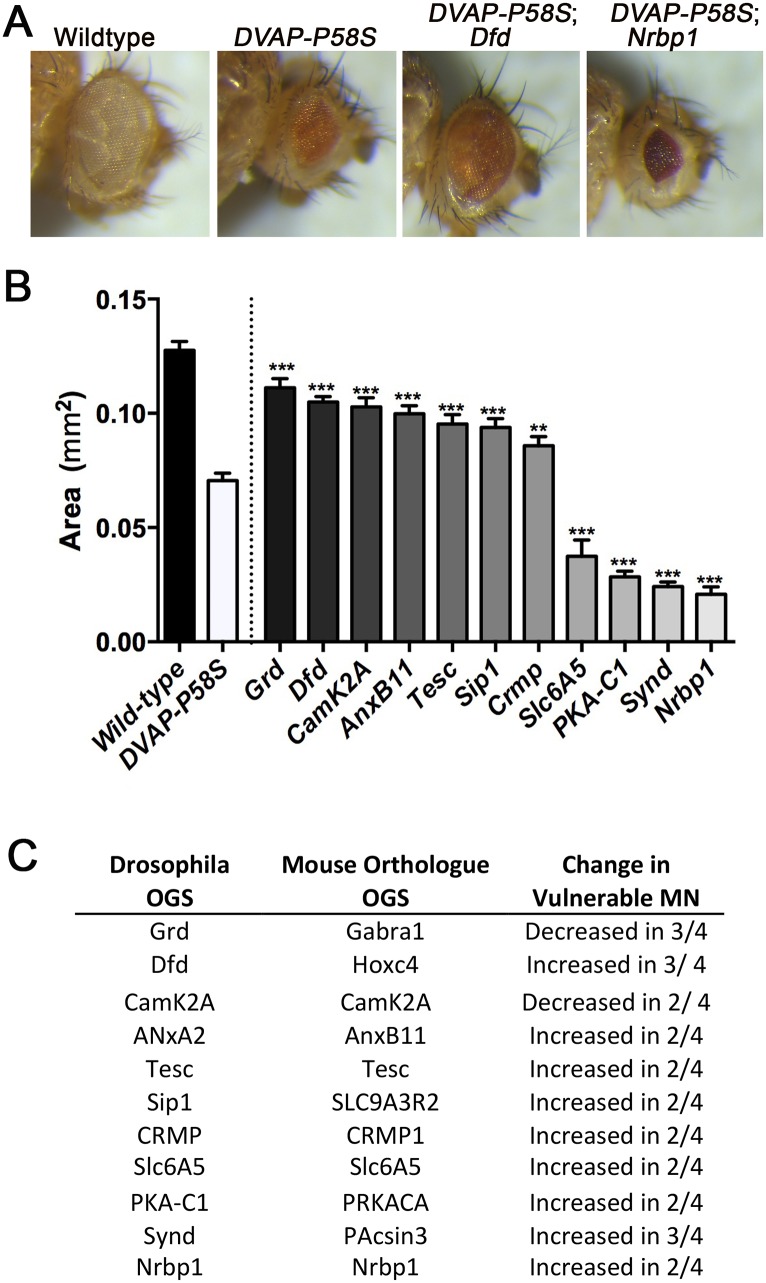
In vivo validation of transcripts in a *Drosophila* model of ALS8. A) Images show eyes from wild-type, DVAP-P58S (carrying ALS8 patient mutation), DVAP-P58S Dfd (AL8 patient mutation with decreased expression of Dfd, the Drosophila homologue of Hoxc4) and DVAP-P58S Dfd (AL8 patient mutation with decreased expression of Nrbp1) Drosophila. Note the decrease in eye size observed in DVAP-P58S flies. This phenotype was supressed by decreased expression of dfd, and enhanced by decreased expression of Nrbp1. B) Bar chart (Mean ± SEM) showing the area (mm^2^) of the eye in *Drosophila* lines which over or under express specified transcripts in DVAP-P58S flies. *** P<0.001, **P<0.01 by ANOVA with Holm-Sidak’s post hoc test. N = approx. 12 flies per group with each value reflecting average of 2 eyes per fly. C) Table details the transcripts which were identified as modifiers of the eye phenotype in DVAP-P58S flies, denoting the Drosphila official gene symbol, the official gene symbol of the mouse homologue and the directional change observed in vulnerable motor neurons in the independent transcriptional screens [16–19]. For those transcripts which were decreased in vulnerable motor neurons, their expression was increased in DVAP-P58S flies, and for those transcripts which were increased in vulnerable motor neurons, their expression was decreased in DVAP-P58S flies.

From this, 66 publically available lines were available to decrease or increase the expression of these transcripts respectively (Table 6). Lines designed to decrease or increase expression our candidate modifiers were crossed with DVAP-P58S flies. Those lines which increase the size of the eye compared to the DVAP-58S flies were categorised as suppressors, and those which decreased the size of the eye were categorised as enhancers of the neurodegenerative phenotype. Overall, 11 transcripts modified the neurodegenerative eye phenotype observed in DVAP-P58S flies, with 7 suppressors and 4 enhancers (Fig 3). Overall, 17% of transcripts displayed an ability of modify the phenotype. Whilst it is difficult to draw direct parallels to other screens, previous screen using *Drosophila* models of motor neuron disease or disease causing mutations, and performing unbiased screen to identifiy modiers have resulted in 0.4 to 4% of genes being identified as enhancers or suppressors. Our increased hit rate compared to these unbiased or enriched suggests that this bioinformatics approach has led to a list which is enriched for disease modifying genes. This suggest that this approach is identifying relevant transcripts which are capable of modifying neurodegenerative pathways associated with motor neuron disease.

**Table 6:** Mouse genes and their Drosophila homologue which were tested in the DVAPP-P58S Drosophila screen.

Mouse symbol	fly symbol	VDRC/ Bloomington Stock number	Mouse symbol	fly symbol	VDRC/ Bloomington Stock number
Up-regulated in vulnerable motor neurons			Down-regulated in vulnerable motor neurons		
**Altered in > 3 screens**					
Hoxb7, hoxd8	Antp	101774	Hdgfrp3	CG7946	22394
Spock1	CG13830	44584	Reps2	Reps	15935
Pygm	CG7180	34369	Rgs8	Dhit	28396
Hoxc4	Dfd	50110	Cbln1	Capr	16833
Fryl	fry	103569	Postn	Fas1	19855
Glra1	GluClalpha	105754	En2	inv	26891
Spsb1	gus	8688	Rev3l	mus205	22360
Hoxa5	Scr	105412			
Pacsin3	Synd	40018, 104580			
Isl2	tup	103585			
**Synaptic or Axonal transcripts altered in >2 screens**					
AnxA2, AnxA6	AnxB10	36107	SLC11A2	Mvl	19886
AnxA2, AnxA6	AnxB11	101313, 29693	SLC7A3	slif	19906
AnxA2, AnxA6	AnxB9	106867	CRMP1	CRMP	40953
ATP1A1	Atpalpha	12330	CAMK2A	CaMKII	22325
LCP1	CG12104	107189	CHL1	CG11403	33544
TESC	CG14362	22733	NCAM1	Fas2	11231
SLC6A5	CG5549	8222	SLC7A3	CG5535	27953
CYB5R3	CG5946	110688	SOCS7	Socs36E	27006
GABRA2	CG8916	9138, 101633			
SLC6A5	DAT	105064, 106961			
GPC1	dlp	29375, 29374			
DCTN2	Dmn	23726, 23728			
LCP1	Fim	6276, 46028			
GNA14	Galphaq	105300			
GNG12, GNG2	Ggamma1	28844			
GABRA5	Grd	5329			
NRP2	Hml	37005, 37006			
ITGA7	mew	44890, 109608			
MMP16	Mmp1	101505			
MMP17	Mmp2	107888			
ABCC3	MRP	7164, 105419			
PRKACA	Pka-C1	101524			
ARHGDIA	RhoGDI	46154, 46155			
KCNA1	Sh	104474, 23673			
SLC9A3R2	Sip1	16108, 16958			
STMN2	stai	977, 13502			

### Over expression of alpha-synuclein modifies phenotype in a mouse model of SMA

**Fig 4:**
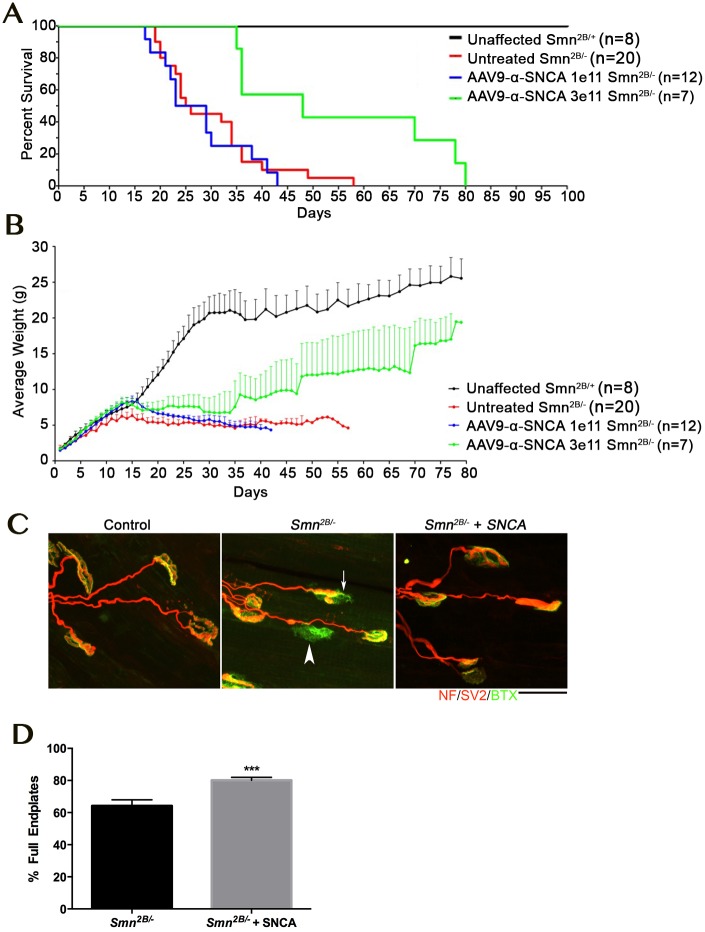
Overexpression of SNCA ameliorate phenotype and neuromuscular junction pathology in the *Smn*^*2B/-*^ mouse model of SMA. A, B) Kaplan Maier plot (A) and weight curve (B) showing profile of control (*Smn*^*2B/+*^; black) and untreated *Smn*^*2B/-*^ (red) compared to mice treated with a low dose (1e^11^; blue) or high dose (3e^11^; green) of AAV9 expressing SNCA. Note that while the low dose only increases weight gain (Student’s *t*-test p < 0.0001, the high dose of AAV9-SNCA significantly increased weight (Student’s *t*-test p<0.0001) and lifespan in the *Smn*^*2B/-*^ mouse model (Mantel-Cox Survival Curve Comparison Test p = 00027). C) Confocal micrographs showing NMJs with the pre-synaptic terminal labeled with antibodies against neurofilament (NF; red) and synaptic vesicle protein 2 (red) and the muscle endplate labeled with alpha-bungarotoxin (green) from the transversus abdominis muscle from P18 mice. Note the presence of fully (arrowhead) and partially (arrow) denervated endplates in the untreated *Smn*^*2B/-*^ mouse which were less commonly observed in the *Smn*^*2B/-*^ mouse treated with high dose AAV9-SNCA. Scale bar = 20μm. D) Bar chart ± SEM showing the increase in the percentage of fully occupied endplates in untreated *Smn*^*2B/-*^ mice (black bars) compared to *Smn*^*2B/-*^ treated with high dose AAV9-SNCA. *** P <0.001 by Mann-Whitney U test where n = 4/8 mice/muscles per group.

Alpha-synuclein (SNCA) was consistently decreased in vulnerable motor neurons across all four screens. This was of particular interest as a decrease in SNCA levels have been reported in SMA patient spinal cord, patient fibroblasts and NSC-34 motor neuron-like cells [29]. There are also a number of studies indicating that, in certain scenarios, over expression of SNCA can be neuroprotective[30–33]. As SNCA is a strong candidate to modify neuronal pathology, we sought to further investigate the effects of SNCA over expression in models of motor neuron disease. Unfortunately, there is no homologue for SNCA, which makes the effect of over expression of SNCA in DVAP-P58S flies difficult to interpret. For this reason we turned to a mammalian model, and sought to determine the impact of SNCA transient expression in the *Smn*^*2B/-*^ mouse model of SMA. To provide widespread expression of the SNCA transgene, an scAAV9-*SNCA* vector was developed. AAV9 has a broad tropism for many tissues within the periphery and the central nervous system, including astrocytes and neuronal lineages [34]. At postnatal day 1, a single injection of 1e^11^ or 3e^11^ viral particles of scAAV9-SNCA was delivered via an intracerebroventricular injection into the *Smn*^*2B/-*^ mouse model of SMA. The lower dose was selected based upon the amount of vector that provides a robust phenotypic rescue using scAAV9-*SMN*[35]. Injection of 1e^11^ viral particles scAAV9-SNCA has no discernable effect on life span or weight gain, however, the higher dose of 3e^11^ viral particles resulted in an ~88% (23 day) increase in median life span and a significant increase in average body weight from approximately P20 onwards (Fig 4A and 4B). Since the initial transcriptomic screen was predicated upon the differential pathology observed at the NMJ, we next examined whether scAAV9-SNCA treatment improved the NMJ phenotype in SMA mice. Importantly, analysis of NMJs from P18 scAAV9-SNCA injected mice revealed a significant increase in the percentage of fully occupied endplates compared to untreated controls, indicative of a decrease in denervation and motor neuron pathology (Fig 4C and 4D).

Together this work demonstrates that in a mouse model of SMA, over expression of *SNCA* can impact upon the neurodegenerative pathways, and has the capacity to extend lifespan and ameliorate the phenotype. This result is a clear proof of principle that this approach can identify relevant phenotypic modifiers that have the capacity to impact disease development in an important model of neurodegeneration.

## Discussion

In this study we have compared multiple screens performed across 3 species to generate a list of common differentially expressed transcripts. We have used a *Drosophila* model to demonstrate that a high proportion of these can modify the neurodegenerative phenotype caused by a human ALS8 mutation. Importantly we have also shown that over expression of *SNCA* can extend life span and decrease NMJ pathology in a mouse models of SMA. This demonstrates that this approach can identify relevant phenotypic modifiers. It also suggests that *SNCA* is an exciting candidate which deserves further attention, and that the remaining candidate list is likely to have some exciting transcripts within it.

The use of transcriptional screen methodologies to dissect the mechanism of disease is central in the study of most pathologies. Screening techniques have been instrumental in identifying affected pathways and perturbed cellular functions. However, when the intended resolution of a transcriptional screen is to identify individual differentially expressed transcripts, it can be difficult to separate those which are changed due to the process of disease, and those which are changed to compensate for a loss of function or due to the altered activity of the organism, organ or cell. Even at pre-symptomatic stages of disease, there are likely to be transcriptional changes occurring as a secondary response to the original pathology. A significant advantage of the current work is to compare the transcriptomes of distinct differentially vulnerable motor neuron populations from normal healthy individuals. This approach allows us to identify those changes which are present in a normal situation, but could potentially impact upon the disease process. Clearly dissecting apart those which are changed by coincidence from those which actually have the potential to modify disease is challenging. This is before the added challenge of identifying those which are actually relevant in humans. However, by comparing across 4 screens, across 3 or more different MNDs, and across 3 species, including humans, we feel we have created an experimental design which has a high chance of identifying clinically relevant modifiers of motor neuron disease.

### Common regulators of vulnerability in motor neuron disease

A number of studies have implicated *SMN1* and *SMN2* copy number in the incidence of sporadic ALS[36]. The observation that ALS causing mutations in *FUS* and *TDP-43* can alter the localisation and associations of Smn has also led to some suggestion of shared mechanism between diseases[36]. Furthermore, although there are certainly some important distinctions in the patterns of selective vulnerability between distinct motor neuron diseases, there are some common themes, in that motor neurons originating in brainstem motor nuclei appear consistently comparatively spared, particularly those supplying the extra-ocular muscles [6, 11–14]. There is therefore good reason to suppose that the mechanisms mediated selective vulnerability in motor neuron disease can, to at least some extent, be shared. In an extension of this, resultant neuroprotective therapies should have the potential to benefit a range of conditions.

The work presented in this study has generated some exciting candidates to be cross-disease modifiers. Aside from *SNCA* (discussed below) there are a number of transcripts which warrant further investigation. CUGBP, elav—like family member 5 (CELF5) belongs to a family of developmentally expressed RNA binding proteins, with a proposed role in pre-mRNA splicing [37]. As this is a function shared by many MND causing mutations, it is easy to generate hypothesis about how differential CELF5 levels may modify pathology. Progesterone receptor membrane component 1 (Pgrmc1) is best characterized due to its role in cancer. However, its oncogenic actions are due in part to its ability to promote cell survival and inhibit apoptosis. PGRMC1 is thought to mediate the protective effects of progesterone on rats modeling Alzheimer’s disease via inhibition of the mitochondrial apoptotic mechanism[38]. It has also shown to be an important mediator in the neuro-protective effect of a synthetic progesterone in the degenerative eye disease retinitis pigmentosa[39]. The observation that Pgrmc1 is decreased in all 4 screens in vulnerable motor neurons could be associated with the increase in cell death of this subpopulation of cells. Stathmin (Stmn1) is a well characterized microtubule binding protein and as such, has important roles in cellular functions dependent upon microtubules, including in mitosis, motility, process formation and intracellular transport[40]. Stathmin has been shown to be dysregulated in a mouse model of ALS, and knockout of stathmin produces a mouse displaying peripheral and central axon degeneration [41, 42]. Interestingly, decreased stathmin levels have previously been shown to increase body weight, motor performance and NMJ maturation is a mouse model of SMA[43]. Therefore amongst our top differentially expressed transcripts we have some very exciting candidates to be modifiers of motor neuron diseases.

### How does SNCA function as a disease modifier

SNCA performs a number of cellular roles, but has been implicated as a causative factor of Parkinsons disease [44]. Mutations in SNCA are strongly associated with aggregate formation, leading to the degeneration of nigrostriatal neurons, causing the well characterised and common disorder of the basal ganglia. Although the pathogenic mechanisms of SNCA aggregates in Parkinson’s is relatively well characterised, the normal function of SNCA is less well defined. It is known to be a small protein of about 140 amino acids localising to the pre-synaptic terminal [45]. It is thought to have an important role in neurotransmitter release. Indeed, over expression of SNCA in primary hippocampal neuron cultures and hippocampal slice culture has been shown to inhibit synaptic vesicle exocytosis, potentially by slowing the recycling of synaptic vesicles and decreasing the number of vesicles in the readily releasable pool[46]. Furthermore, alpha-synuclein knockout mice display an increased rate of vesicle filling under repetitive stimulation[47]. How then might this role of SNCA be a protective modifier in motor neuron diseases? The idea of SNCA possessing neuroprotective qualities is not novel. Whilst some have suggested that over expression of wild-type SNCA could increase vulnerability to certain insults such as oxidopamine (a toxin specific to dopiminergic neurons)[33], other work has shown that increased SNCA can decrease toxicity caused by the pesticide paraquat [32], the apoptotic ages staurosporin and etoposide [30] and oxidative stress induced by hydrogen peroxide [31]. The resistance to oxidative stress observed was proposed to be due to a down regulation of the cell stress induced c-Jun N-terminal kinase pathway which promotes apoptosis[31]. The mechanism by which SNCA could be a protective modifier in the context of this study is currently unclear. However given the multiple scenarios in which SNCA has been shown to be neuroprotective, further work is justified to explore this mechanism.

### Why are transcripts differentially expressed in motor neurons?

As the screens detailed in this report have been performed in exclusively healthy motor neurons, which happen to be differentially vulnerable in disease, the transcriptional changes which we are reporting likely occur for reasons unrelated to motor neuron pathology. It is therefore important to consider why the transcriptional changes exist. These transcriptional changes may reflect differences in the development, function, physiology or anatomy of the individual motor neurons. For example, we might suggest that cranial motor neurons generally have a shorter axonal length that those located elsewhere in the body. It is also possible that they have have other structural differences such as a more elaborate dendritic tree, or a different proportion of axodendritic or axosomatic synpases. The potential differences in form and function between motor neuron pools are seemingly endless. We can also only currently hypothesize about how changes in development, form or function could result in a selective sparing in a pathological situation. For this reason, rather than dismissing transcriptional changes occurring between differentially vulnerable motor neuron as, most likely due to a difference in the development, location or function of a given pool of motor neurons, it may be useful to use the list of transcripts identified to generate ideas about how this can impact upon the anatomy and physiology of the motor neuron. Indeed, the observation that a large number of HOX genes were differentially expressed between differentially vulnerable motor units may be attributed to the different location of the different motor neuron pools. However, it may be that the actual difference in location, and the associated differences in anatomy and physiology actually contribute to their differentially vulnerabililty. Determining the reasons and consequences of the differential transcriptional expression may lead to to a broader understanding of what fundamental differences make a motor neuron more or less vulnerable during disease. We therefore suggest that future efforts, to understand the impact of differential expression of specific transcripts or alterations in specific cellular pathways relate to the development, physiology and anatomy of specific motor neuron pools may be fruitful in our search to understand the phenomenom of selectively motor unit vulnerability of motor neuron disease.

### Conclusions

In this work we have employed a novel approach to identify transcripts that are functionally significant in motor neuron disease-relevant pathways. We have demonstrated that at least one of these candidates can modify the phenotype in a mouse model of SMA, and believe that the remaining list contains additional candidates that warrant further examination. Based upon the design of the experiments, these modifiers may functionally interact in more than one disease context and therefore have the ability to provide protection to motor neurons in a variety of neurodegenerative conditions. Future efforts to identify potent modifiers and their mechanisms of action will provide insight into the mechanism of disease, and aid in the development of therapeutic agents which can slow the degeneration of motor neurons in MND.

## Materials and methods

### Data acquisition and analysis

RNAseq data, profiling the transcriptome of cranial and abdominal motor neurons from P10 wildtype mice was obtained as detailed in Murray *et*. *al*.[16]. Further analysis of this data was allowed by generous agreement with Dr Rashmi Kothary. The raw microarray files detailing transcriptional data published in Kaplan et al., 2014 and Brockington et al., 2013 were downloaded from the gene expression omnibus using the reference numbers GSE52118 and GSE40438 respectively[17, 19]. Raw microarray files from the study by Hedlund et al. were generously provided by Dr Eva Hedlund[18].

Following acquisition of raw microarray data sets, data was normalised using a quantile method, and genes which were differentially expressed within each screen were identified. All genes with an adjusted P value of >0.05 were eliminated from the study. For RNAseq data, transcripts had been identified by alignment to the mouse mm9 genome assemble in Murray *et*. *al*.[16], and relative transcript levels were compared using CuffDiff software v1.3 using the UCSC transcript model. Significance was considered with an adjusted P value of <0.05 and a greater that 1.5 fold change in expression level. HomoloGene was use to identify the genetic homologue between species. Data was sorted in excel to reveal changes which occurred in a common direction in 2 or more screens.

### Screening methods and *Drosophila* husbandry

Genetic schemes and *Drosophila* husbandry were performed as detailed in Sanhueza et al., 2015[23]. Briefly, the tester line carrying both the *ey-Gal4* driver and the *UAS-DVAP-P58S* transgene on the second chromosome was crossed individually with RNAi and EP lines with the potential of overexpressing the gene of interest. The F1 progeny was analyzed for the suppression or the enhancement of the DVAP-P58S induced small and rough eye phenotype. In particular, 8–10 males of either the EP or RNAi line were mated to 10–15 females of the *ey-Gal4*, *DVAP-P58S/CyO* ALS8 fly stock. After two days, adults were transferred to a new vial to have a duplicate cross. Embryos from both vials were raised at 29°C in a water bath to maximize the effect of the Gal4. Both enhancing and suppressing effects of the DVAP-P58S-induced eye neurodegenerative phenotype were assessed in these conditions. RNAi lines were acquired from the Vienna *Drosophila* RNAi line Center while EP and EPgy lines were obtained from the *Drosophila* Bloomington Stock Center.

### Mouse maintenance

For analysis of differentially vulnerable muscles, *Smn*^*2B/-*^ mice and wildtype controls on a C57Bl6 background were maintained in the animal facilities at the University of Edinburgh. Mice were sacrificed by overdose of inhalation anaethetic (isofluorane) and cervical dislocation. All experiments were performed in accordance with the regulations set out by the UK Home Office. For experiments requiring the administration of AAV9, all mice were housed and handled in accordance with the Animal Care and Use Committees of the University of Missouri. *Smn*^*2B/2B*^ mice were a kind gift from Dr. R. Kothary (Ottawa, Canada). FVB *Smn*^*+/-*^ mice were purchased from the Jackson Laboratory. Mice were housed under a 12 hours light/dark cycle and the colonies were maintained as heterozygote breeding pairs under specific pathogen-free conditions.

### Vector construction

293T HEK cells (ATCC CRL 3216, American Type Culture Collection, Manassas, VA, USA) cultured in 4 10-floor cell factories until ~85% confluent. Cells were triple transfected with Rep2Cap9 (Serotype 2 Rep proteins, Serotype 9 capsid proteins), pHelper (Adenovirus helper constructs), and scAAV-CBA-*SNCA* using 25 kDa Polyethyleneimine (PEI) at a molar ratio of 1:1:1. Media was changed 24 hours after transfection, and cells were harvested at 48 hours after transfection. Cells were suspended in 10 mmol Tris, pH = 8.0, lysed by 5 freeze-thaw-cycles in liquid nitrogen, DNAse treated, and protease treated. CsCl crystals were added to the lysate (0.631 g of CsCl per ml of the lysate) to generate a solution with a density of ∼1.4 mg/ml. After incubation at 37°C for 45 min, the solution was centrifuged at 4000 rpm in an Eppendorf 5810 R at 4°C. Virus was purified from lysate by 3 rounds of density gradient centrifugation at an average RCF of 158,000. High titer fractions were detected after each round of centrifugation using quantitative real-time PCR. The final fractions were dialyzed exhaustively against phosphate buffered saline and stored at 4°C until use.

### Administration of AAV9 vectors

Viral delivery was performed by intracerebroventricular (ICV) injection using methods described previously [48, 49]. Briefly, ICV injections were performed using sterilized glass micropipettes. The needles were inserted perpendicular to the skull at the injection site approximately 0.25 mm lateral to the sagittal suture and 0.5 mm rostral to the coronary suture.

### Immunofluorescent staining and quantification

For NMJ labelling, muscles were immediately dissected from recently sacrificed mice and fixed in 4% PFA (Electron Microscopy Science) in PBS for 15 min. Post-synaptic AChRs were labelled with α-bungarotoxin (BTX) for 30 min. Muscles were permeabilised in 2% Triton X-100 in PBS for 30 min, then blocked in 4% bovine serum albumin (BSA)/1% Triton X-100 in PBS for 30 min before incubation overnight in primary antibodies [Neurofilament (NF; 2H3)—Developmental Studies Hybridoma Bank; synaptic vesicle protein 2 (SV2)—Developmental Studies Hybridoma Bank; S100 –Dako; all 1:250] and visualised with Cy3-conjugated secondary antibodies [Cy3 goat anti-mouse; 1:250, Jackson]. Muscles were then whole-mounted in Dako Fluorescent mounting media. Confocal microscopy was performed using a Nikon A1R^+^ Resonant Scanning System (Nikon) (10x and 40x objectives; 0.3 and 1.3 oil NA; Nikon A1R^+^ microscope; simultaneous image acquisition). 488 and 543 nm laser lines were used for excitation. The resultant confocal Z-series produced in NIS Elements 2D Analysis software were exported and merged using Fiji ImageJ software.

The percentage of fully occupied endplates was determined by classifying each endplate in a given field of view either fully occupied (pre-synaptic terminal (SV2 and NF) completely overlies endplate (BTX)), partially occupied (pre-synaptic terminal only partially covers endplate (BTX)), or vacant (no pre-synaptic label overlies endplate). At least 4 fields of view were analysed per muscle totalling >100 endplates per muscle.

All data was assembled and analysed using Microsoft Excel and GraphPad Prism.

## Supporting information

S1 Table Results from RNAseq comparing vulnerable (innervating the abdominal muscles) and reistant (innevating the cranial muscles) motor neurons from P10 WT mice. Table shows the ensembl ID (test-ID, gene_id), the official gene symbol (gene), the chromosomal location (locus), the average normalised read count from an N of 2 samples for resistant (BS-WT) or vulnerable (SC_WT) samples, the log2 fold change, and the relevant statistics (test-stat, P_value, Q-value, significant).
(XLSX)

S2 Table Transcriptional changes which occurred in 3 of more of the included transcriptional screens. Table includes the mouse official gene symbol and the log2 fold change identified in Brockington et al., Kaplan et al., Murray et al., and Hedlund et al.
(XLSX)

S3 Table Transcriptional changes which occurred in 2 of more of the included transcriptional screens. Table includes the mouse official gene symbol and the log2 fold change identified in Brockington et al., Kaplan et al., Murray et al., and Hedlund et al.
(XLSX)
